# Supplementary material for: Identification of photoperiod-induced specific miRNAs in the adrenal glands of Sunite sheep (Ovis aries)
Source: Front Vet Sci. 2022 Jul 22;9:888207. doi: 10.3389/fvets.2022.888207 (PMC9354845; doi:10.3389/fvets.2022.888207)
Supplement: Supplementary file 1 [file Data_Sheet_1.ZIP › Supplementary file/miRDeep2 file.zip!/miRDeep2 file/Novel_Structure/LP42A3/Novel_687.pdf]

Provisional ID	:	NC_019462.2_8781
Score total	:	3.5
Score for star read(s)	:	3.9
Score for read counts	:	-3.3
Score for mfe	:	1.5
Score for randfold	:	1.6
Score for cons. seed	:	
Total read count	:	5
Mature read count	:	4
Loop read count	:	0
Star read count	:	1

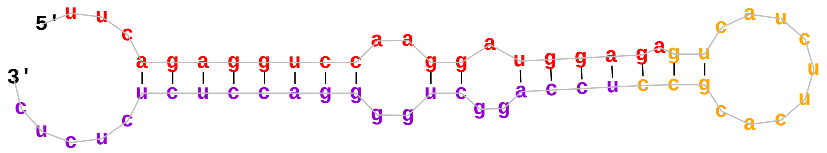

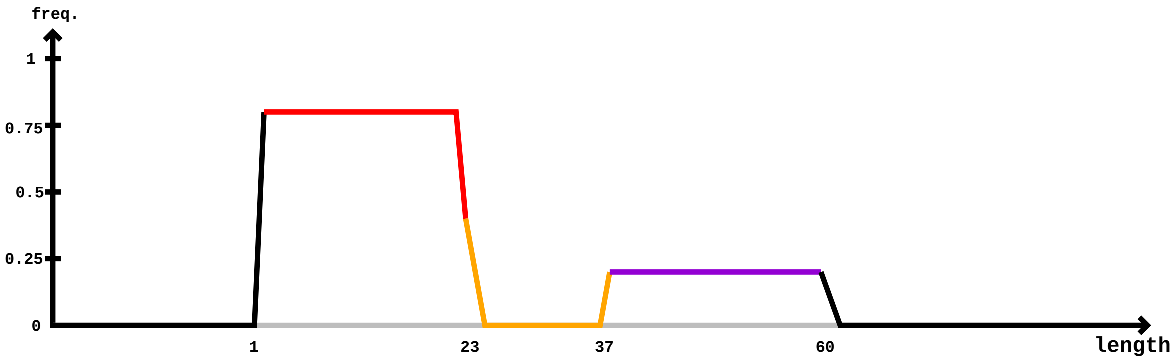

Mature **Star**

```
5'- ugacuaaagagaugggacgauucagagguccaaggauggagagucaucuucacgccuccaggcuggggaccucucucucagcccucucauuucugugcuucuccacccaccc  -3'   obs
    ugacuaaagagaugggacgauucagagguccaaggauggagagucaucuucacgccuccaggcuggggaccucucucucagcccucucauuucugugcuucuccacccaccc        exp
    ........((((.(((..((...((((((((..((.(((((.((.........)))))))..))..))))))))...))..)))))))......(((......)))......   reads   mm   sample
    ....................uucagagguccaaggauggag.......................................................................   2       0    seq
    ....................uucagagguccaaggauggaga......................................................................   2       0    seq
    ........................................................uccaggcuggggaccucucucuc.................................   1       0    seq
```
